# Application of a convolutional neural network for predicting the occurrence of ventricular tachyarrhythmia using heart rate variability features

**DOI:** 10.1038/s41598-020-63566-8

**Published:** 2020-04-21

**Authors:** Getu Tadele Taye, Han-Jeong Hwang, Ki Moo Lim

**Affiliations:** 1 Health Informatics Unit, School of Public Health, Mekelle University, Mekelle, Ethiopia; 2 Department of Electronics and Information Engineering, Korea University, Sejong, 339-770 Korea; 3 Department of IT Convergence Engineering, Kumoh Institute of Technology, Gumi, South Korea

**Keywords:** Computational models, Machine learning

## Abstract

Predicting the occurrence of ventricular tachyarrhythmia (VTA) in advance is a matter of utmost importance for saving the lives of cardiac arrhythmia patients. Machine learning algorithms have been used to predict the occurrence of imminent VTA. In this study, we used a one-dimensional convolutional neural network (1-D CNN) to extract features from heart rate variability (HRV), thereby to predict the onset of VTA. We also compared the prediction performance of our CNN with other machine leaning (ML) algorithms such as an artificial neural network (ANN), a support vector machine (SVM), and a k-nearest neighbor (KNN), which used 11 HRV features extracted using traditional methods. The proposed CNN achieved relatively higher prediction accuracy of 84.6%, while the ANN, SVM, and KNN algorithms obtained prediction accuracies of 73.5%, 67.9%, and 65.9% using 11 HRV features, respectively. Our result showed that the proposed 1-D CNN could improve VTA prediction accuracy by integrating the data cleaning, preprocessing, feature extraction, and prediction.

## Introduction

Heartbeat is regulated by electrical signals conducted across the four chambers of the heart: two atria and two ventricles. When electrical activity is normal, the heart beats approximately 60 to 100 times per minute. However, abnormal electrical signals in the heart lead to disorganized electrical activities such as ventricular tachyarrhythmia (VTA), which causes fast heart rate^1^. Thus, early VTA prediction helps physicians to take immediate medical procedure to reduce the risk. Ventricular tachycardia (VT) and ventricular fibrillation (VF) are the most common VTAs. VT arises from improper electrical activity in the ventricles, and can cause sudden cardiac arrest. VF is caused by chaotic electrical activity in the ventricles, which is similar to VT, but is a fatal condition that requires immediate medical attention. In VF, the heart shivers instead of pumping blood.

Developing earlier preventive interventions would reduce the risk of experiencing an imminent VT and VF events. Researchers used noninvasive tests by measuring and analyzing electrocardiograms (ECGs), where heart rate variability (HRV) is extracted to train machine learning (ML) algorithms for predicting VT or VF in advance^2^. HRV is the most commonly employed biomarker for isolating VT or VF subject from the normal subject^3^. It is a time variation of heartbeats among two successive QRS complexes (Q, R, and S waves in ECG). In recent years, HRV indices have been used as a noninvasive biomarkers to forecast life-threatening arrhythmias^4^. Previous studies mainly used the three traditional analysis methods: time domain, frequency domain, and Poincare nonlinear analyses, to extract features from HRV. Furthermore, they used these features as input to machine learning algorithms to predict the occurrence of VT, VF, or both. The machine learning techniques are used to classify the complex feature patterns and enable early prediction of VT or VF events with high accuracy.

Acharya *et al*. used features extracted from HRV for classifying cardiac disorders by applying artificial neural network (ANN) and fuzzy equivalence relations^5^. In their study, cardiac disorders were classified into four categories: ischemic/dilated cardiomyopathy, complete heart block, atrial fibrillation (AF), and normal sinus rhythm. Bilgin *et al*. used HRV to study the feasibility of sub-band frequency analysis to predict VTA, and compared the traditional frequency analysis using low frequency (LF) and high frequency bands^6^. They used multilayer perceptron (MLP) neural network to evaluate the sub-bands of LF and HF obtained using wavelet packet transform (WPT). Elias *et al*. performed time-frequency and Poincare nonlinear analyses to extract HRV features and evaluated the SCD prediction performance of their methods in patients with sustained VTA^7^. Successive one-minute intervals such as first, second, third, and fourth minutes before the event were used for extracting features. Then MLP and k-nearest neighbor (KNN) algorithms were used to predict the event^7^. Joo *et al*. extracted HRV features and applied an ANN to predict the occurrence of VT and VF 10 s in advance^8^. They extracted the features using the aforementioned three traditional analysis methods. In our previous study, we investigated the feasibility of QRS complex shape features on VF onset prediction, where we used ANN and demonstrated the superiority of QRS features as compared to traditional HRV features in terms of VF prediction performance^9^.

The methods adopted by previous studies were sensitive to noise; they therefore required data cleaning, and they involved separate dataset preprocessing, feature extraction, and prediction. Our study proposes a prediction method using one-dimensional convolutional neural network (1-D CNN) that contains all aforementioned processes together. With the advent of deep learning, 1-D CNN has become favorable for extracting features from time series signals, and therefore for detection, prediction, and classification^10^. The essential part of this approach is that data cleaning is not required, and the feature extraction and prediction do not need to be explicitly defined^11^. Thus, deep learning represents the learning process that consists of an input layer, hidden layers, and an output layer^12^. It is representation-based learning that learns features from raw data using a series of layers for prediction. Therefore, CNN possesses the capacity to extract features from the 1-D time series data of raw ECG signals and use them to monitor mental stress and detect myocardial infractions (MI)^11^. However, no studies have used CNN with HRV for predicting imminent VT or VF.

We propose a prediction method for VT and VF based on a 1-D CNN trained using HRV signals. Our goal is to predict the onset of VT and VF within 1 min by extracting features from 5 min HRV signals.

## Results

Statistical differences of HRV features of VTA datasets and control datasets were observed before we trained ANN and the other ML algorithms. Table 1 shows the comparison of the means and standard deviations of the HRV features between the control and VTA datasets. Five of the eleven features – mean NN (MRRI), VLF, LF/HF, SD2, and SD1/SD2 – show statistically significant differences between the two groups (two tailed t-test, *p* < 0.05).

**Table 1:** Comparison of HRV features between control and VTA dataset. The means and standard deviation were calculated after removing some outliers (LF/HF > 10).

Features	VTAs dataset (n = 135)	Control dataset (n = 126)	p-Value
	Mean ± SD	Mean ± SD	
Mean NN(MRRI) (ms)	684.45 ± 125.47	775.15 ± 107.61	<0.001
SDNN (ms)	74.34 ± 49.08	79.04 ± 48.08	0.07
RMSSD (ms)	48.68 ± 43.32	47.06 ± 40.13	0.5
pNN50 (%)	18.18 ± 19.93	16.13 ± 19.43	0.05
VLF	1070.61 ± 2591.31	1331.09 ± 2069.39	0.03
LF	3453.29 ± 22523.1	3766.81 ± 18825.98	0.8
HF	13286.95 ± 92857.74	8931.81 ± 73327.59	0.3
LF/HF	0.998 ± 1.35	1.774 ± 2.19	<0.001
SD1	34.42 ± 30.63	33.28 ± 28.38	0.48
SD2	98.05 ± 64.29	105.53 ± 63.8	0.03
SD1/SD2	0.35 ± 0.16	0.31 ± 0.16	<0.001

Features extracted using CNN had the highest prediction performance of 84.6%. Using elven traditional HRV features; the KNN, SVM, and ANN achieved prediction accuracies of 65.9%, 67.9%, and 73.5%, respectively (Table 2). We used neural network with two hidden layers which contain 22 neurons each showed the best prediction performance.

**Table 2:** The results for the CNN in predicting VTA 60 s before its occurrence.

Algorithms	Sensitivity (%)	Specificity (%)	Accuracy (%)	AUC
KNN	64.3	68.2	65.9	0.62
SVM	67.9	68.6	67.9	0.63
ANN	69.9	78.2	73.5	0.65
CNN	83.2	86.4	84.6	0.78

The means and standard deviations of the prediction accuracies evaluated using 10-fold cross validation for all algorithms. Figure 2A shows the prediction accuracies of the CNN, ANN, SVM, and KNN algorithms. The single asterisk (*) shows a statistically significant difference among the algorithms. Thus, prediction accuracies of the CNN, ANN, SVM, and KNN algorithms yielded statistically significant differences (one-way ANOVA: F(3, 36) = 27.38, *p* < 0.001); a post-hoc test showed that the CNN statistically outperformed the other algorithms (*p* < 0.001). However, the prediction accuracies of the ANN, SVM, and KNN were not significantly different (*p* > 0.05).

To observe how much our model is capable of distinguishing between VTA and healthy subjects, we depicted ROC curve. Figure 2B shows the ROC curves of the CNN, ANN, SVM, and KNN algorithms. The CNN showed the highest AUC (0.78) as compared to the ANN (0.65), SVM (0.63), and KNN (0.62).

## Discussion

In this study, we proposed a CNN algorithm to predict the onset of an imminent VTA using HRV signal, and the CNN algorithm showed the highest prediction accuracy (84.6%) compared to other machine learning algorithms (KNN, SVM, and ANN). The CNN algorithm used in this study was adopted from Acharya *et al*.’ study that used a 1-D CNN to perform feature extraction and selection together to detect myocardial infraction (MI) using ECG signals^11^. Though our target disease was different from the target disease used in Acharya *et al*.’s study, the same 1-D CNN model worked very well to predict the occurrence of VTA in advance.

Previous studies mainly dealt with traditional feature extraction methods using HRV signals and VTA prediction using either classical machine learning algorithms or an ANN. An ANN proposed by Joo *et al*. demonstrated accuracy of 75.6% for predicting VTA 10 s before onset^8^. Lee *et al*. increased the forecast time to 1 h and predicted the occurrence of VT with an accuracy of 73.5% using traditionally extracted HRV features^1^. Similarly, Melillo *et al*. used several machine learning algorithms, such as the random forest (RF), SVM, and MLP to identify hypertensive patients at high risk using HRV signal. The accuracies for the RF, SVM, and MLP algorithms were 85.7%, 83.9%, and 76.8%, respectively. The accuracies for the RF and SVM are similar to the accuracy of our CNN, although direct comparison of the results was not appropriate because the datasets were from different cases^13^.

In our previous study^9^, we proposed a novel feature type driven from QRS complex shape to improve the performance of VF onset prediction, where we used ECG and HRV datasets from the PhysioNet repository, but the HRV dataset was different from that used in this study. In the previous study, we compared the VF prediction performance obtained using QRS features with that obtained using traditional HRV features, and demonstrated the superiority of the proposed QRS features in terms of VF prediction performance. The fundamental goal of the previous study was to investigate the feasibility of the new QRS features on the improvement of VF prediction performance while the goal of this study was to verify the usefulness of the CNN algorithm on feature extraction and VTA prediction.

All the aforementioned studies used the traditional method which requires separate data cleaning, preprocessing, feature extraction, and classification. However, our CNN does not require data cleaning and combines feature extraction and classification, which makes it more favorable to traditional machine learning algorithms^11^.

Our proposed algorithm had drawbacks; it was computationally expensive to train, and it required a large dataset. To overcome the limitation, we applied data augmentation to increase the size of our dataset, however it was still small to train our CNN. To implement this study for clinical application, a large dataset is required for training. Once the algorithm is trained, the system can immediately predict the occurrence of VTA. Our algorithm is generalizable because it did not encounter performance degradation on the new inputs from the same distribution of the training dataset. Generalizability is strongly related to the concept of overfitting. If a model is overfitted then it will not generalize well. To overcome overfitting, we used 10-fold cross validation, and our algorithm performed well on new unseen data.

Our algorithm could be installed in patients’ implantable cardiac defibrillations (ICDs) for real-time VF prediction as additional functionality to VF detection. Our algorithm could also be affected by false detections. When FN occurs, the test could be performed once again by the CNN algorithm (this could be detected at the time of occurrence, not the prediction time before the onset). However, FP could lead to wrong ventricular defibrillation by the ICD, which may have a fatal effect on the patient. Therefore, further investigation should be continued to minimize false detection in the future. Predicting the occurrence of VF hours in advance would be more useful, but the dataset used for this study were limited to a 5 min data window, and predict VF 1 min before its occurrence.

## Methods

### Dataset

The dataset was collected from a database in PhsyioNet known as spontaneous ventricular tachyarrhythmia database (MVTDB)^14^, which consisted of 135 pairs of RR interval time series recorded by implantable cardioverter defibrillators (ICD) (Medtronic Jewel PlusTM ICD 7218) in 78 patients (as shown in Table 3: 63 male and 15 female, aged 20.7–75.3, mean weight: 79.82 kg, and mean height: 172.62 cm). Note that each patient had different numbers of VF and VF events. Each pair of the dataset included VT or VF and its corresponding normal sinus rhythm (control) from which we extracted 106 VT, 29 VF, and 126 control datasets (there were 135 datasets but 9 datasets were duplicated). The sampling frequency of the ECG, from which the RR intervals were collected, was 1000 Hz.

**Table 3:** Subject characteristics.

Variables	Subjects (78)
Age (Years)	20.7–75.3
Average Height (cm)	172.62
Average Weight (Kg)	79.82
Number of Male	63
Number of Female	15

### Preprocess

All HRV signals collected form PhysioNet were cut starting from the onset of the VTA which was considered as 0 s to 360 s before the onset. Then, we divided the 360 s long signal into two parts: required time and forecast time. The required time represents the time period used for feature extraction between 360 and 60 s before the VTA onset time. The forecast time is the time period between 60 and 0 s before VTA onset. Using the required time data, we could predict the occurrence of VTA before the forecast time. Because the number of RR intervals laid in the required time had different sample points ranging from 314 to 883 RR sample points, we decided to resample the data, so all dataset would have the same RR sample points within the require time period. The reason for having the same RR sample points is that the CNN requires the same input size. Therefore, we used interpolation to resample our data to have 1000 RR sample points as input to our CNN algorithm.

We applied data augmentation to increase the number of the datasets by applying circular shift on the RR intervals using randomly generated integer values. We used roll() function from Numpy to roll the sample points, and elements that roll beyond the last position are re-introduced at the first. Circular shift obviously changes the order of the RR sample points and hence change the features. We observed that the HRV features after data augmentation had values within the range of the means and standard deviations of the features before data augmentation, Table 1 shows the means and standard deviations after augmentations. Therefore, the VTA dataset remains VTA dataset and control remains control after data augmentation. The dataset size increased from 261 to 1566, in total.

## Feature Extraction

### Traditional feature extraction method

We extracted features from the required time region, between 360 s and 60 s before VT and VF occurs. Eleven HRV features were extracted, among which four are time domain features, four are frequency domain features, and three are Poincare nonlinear features. Among the four frequency domain analysis features, we observed LF/HF ratio values of each dataset to check for outliers. Nunan *et al*.^15^ and Pikkujamsa, Sirkku M., *et al*.^16^ suggested the possible ranges of LF/HF ratio for healthy individuals (the control) to be from 1.1 to 11.6. Therefore, we decided to remove values greater than 11.6, which comprised 174 datasets. The total dataset size for our study became 1392 recordings.

### Time domain analysis

Four HRV features were computed in this category^1,7^: Eq. (1) mean RR intervals (Mean NN (RR)), Eq. (2) standard deviation of NN (RR) intervals (SDNN), Eq. (3) square root of mean squared difference of successive NN (RR) intervals (RMSSD), and Eq. (4) the proportion of interval differences of successive NN (RR) intervals greater than 50 ms by the total number of NN (RR) intervals (pNN50), defined as follows:

1 $$MeanNN=1/N\sum RR(i),$$

2 $$SDNN=\sqrt{1/N\sum (RR(i+1)-MeanNN){)}^{2},}$$

3 $$RMSSD=\sqrt{1/N{\sum (RR(i+1)-RR(i))}^{2}},$$

4 $$pNN50=\frac{|RR(i+1)-RR(i)| > 50ms}{total\,Number\,of\,RR\,intervals}\times 100.$$

### Frequency domain analysis

The datasets were corrected by removing the DC offset before frequency domain analysis was performed. We considered three frequency bands: the very low frequency (VLF) band (0–0.04 Hz), low frequency (LF) band (0.04–0.15 Hz), high frequency (HF) band (0.15–0.4 Hz), as well as the ratio of LF and HF. We computed the power spectrum density (PSD) of the bands using Welch’s periodogram with a Hanning window (window size: 256 points with an overlap of 50%).

### Poincare nonlinear analysis

The Poincare nonlinear features were dispersion of points perpendicular to and points along the axis of the line-of-identity^1,7^. The standard deviation of the successive RR intervals scaled by $1/\sqrt{2}$ (SD1) and the standard deviation of points along the axis of the line-of-identity (SD2) were both calculated using Eqs. (5) and (6). We considered the ratio of SD1 and SD2 as well.

5 $$SD1=\sqrt{\frac{1}{2}\,Var(RR(i)-RR(i+1))},$$

6 $$SD2=\sqrt{2SDN{N}^{2}-\frac{1}{2}\,SD{1}^{2}}.$$

### Feature extraction using CNN

We extracted feature from HRV signals using the 1-D CNN model to predict the VTA onset, and compared the prediction performance with the traditional feature extraction method. The proposed 1-D CNN model is comprised of an input layer followed by four convolutional layers, batch normalization, and pooling layers. One convolution layer is defined by filters (F), strides (S), and padding (P), which used rectified linear unit (RELU) activation functions. These layers were built using different configurations: the first convolution layer has three filters of size 102, the second has ten filters of size 24, the third has ten filters of size 11, and the fourth has ten filters of size 9. After each convolution layer, batch normalization was used to accelerate the CNN training by reduction the dependency gradients on the scale of the features.

A pooling layer follows the batch normalization to reduce the dimensionality of the feature map without affecting the important features^17^. We used maxpooling as a down-sampling strategy that chooses the maximum values in the vector outputs from a convolutional filter. Finally, the convolutional and pooling layers perform the feature extraction^10^, their output is flattened to a 1-D tensor (fully connected layer) and fed to a dense layer for prediction^18^.

**Figure 1:**
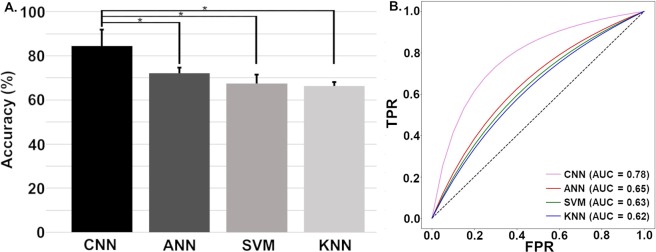
(**A**) CNN architecture with an input layer, four hidden layers, and a flatten input that will be fed to dense layers. (1D: one dimension) (**B**) The architecture of our ANN.

As shown in Fig. 1A, the CNN contained an input layer with 1000 neurons (sample points), twelve hidden layers grouped into four stacks of convolution, batch normalization, and maxpool layers. After flattening the last output, the classification network was constructed using three dense layers, to form end-to-end structure in which classification and feature extraction merged seamlessly.

**Figure 2:**
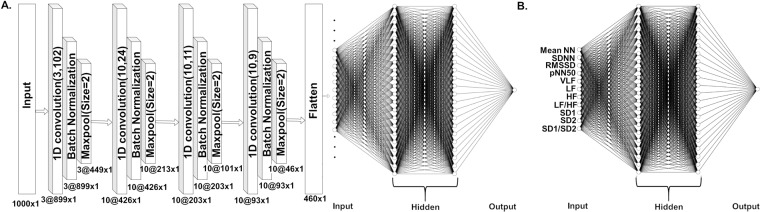
(**A**) Means and standard deviations of the prediction accuracies of each algorithm. Single asterisks (*) indicates a statistically significant difference between the prediction accuracies of different algorithms (CNN > ANN, SVM, and KNN, *p* < 0.001). (**B**) ROC AUCs (receiver operating characteristic area under curves) of CNN, ANN, SVM, and KNN to predict VTA 60 seconds before the occurrence. TPR: True Positive Rate and FPR: False Positive Rate accuracies.

### Prediction algorithms

We used similar neural networks to predict VTA for CNN features (classification process of Fig. 1A) and eleven traditional HRV features (Fig. 1B) to fairly investigate the impact of different feature extraction methods. The classification part of the CNN in Fig. 1A has the same number of layers as the ANN model in Fig. 1B. Both contained nodes to help capture nonlinearity in the input data, and an output layer, which contained a node to represent a dependent variable (VTA occurrence)^19,20^. We used rectified linear unit (RELU)^21^ activation functions for the hidden layers, and the sigmoid activation function^22^ for the output. The two hidden layers consisted of 22 neurons each. We attained the ideal network for the dataset by repeated trial experimentation. We also implemented the support vector machine (SVM) and k-nearest neighbors (KNN) algorithms to compare their prediction performances with our proposed CNN algorithm. We applied 10-fold cross validation^23^ for each algorithm to avoid overfitting. The dataset was randomly divided into approximately ten groups, one group was used as the testing dataset, while the remaining nine groups were used for training.

In this study, we attempted to demonstrate the performance of a CNN to predict the occurrence of VTA using 5 min HRV signals, 1 min in advance. The amount of the dataset used in this study was relatively small for training the CNN algorithm, and thus we applied data augmentation to increase the size. The proposed CNN algorithm yielded a prediction accuracy of 84.6%, which is higher than the other machine learning algorithms. Furthermore, this study requires further investigation using a greater number of datasets for clinical application.
