# Author Correction: Application of a convolutional neural network for predicting the occurrence of ventricular tachyarrhythmia using heart rate variability features

**DOI:** 10.1038/s41598-020-68530-0

**Published:** 2020-07-08

**Authors:** Getu Tadele Taye, Han-Jeong Hwang, Ki Moo Lim

**Affiliations:** 1 Health Informatics Unit, School of Public Health, Mekelle University, Mekelle, Ethiopia; 2 Department of Electronics and Information Engineering, Korea University, Sejong, 339-770 Korea; 3 Department of IT Convergence Engineering, Kumoh Institute of Technology, Gumi, South Korea

Correction to: *Scientific Reports* 10.1038/s41598-020-63566-8, published online 21 April 2020

This Article contains an error in the order of the Figures. Figures 1 and 2 were published as Figures 2 and 1 respectively. The Correct Figures 1 and 2 appear below. The Figure legends are correct.

**Figure 1:**
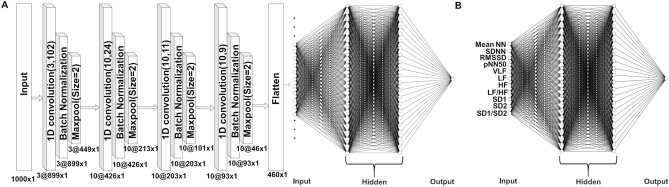
(**A**) CNN architecture with an input layer, four hidden layers, and a flatten input that will be fed to dense layers. (1D: one dimension) (**B**) The architecture of our ANN.

**Figure 2:**
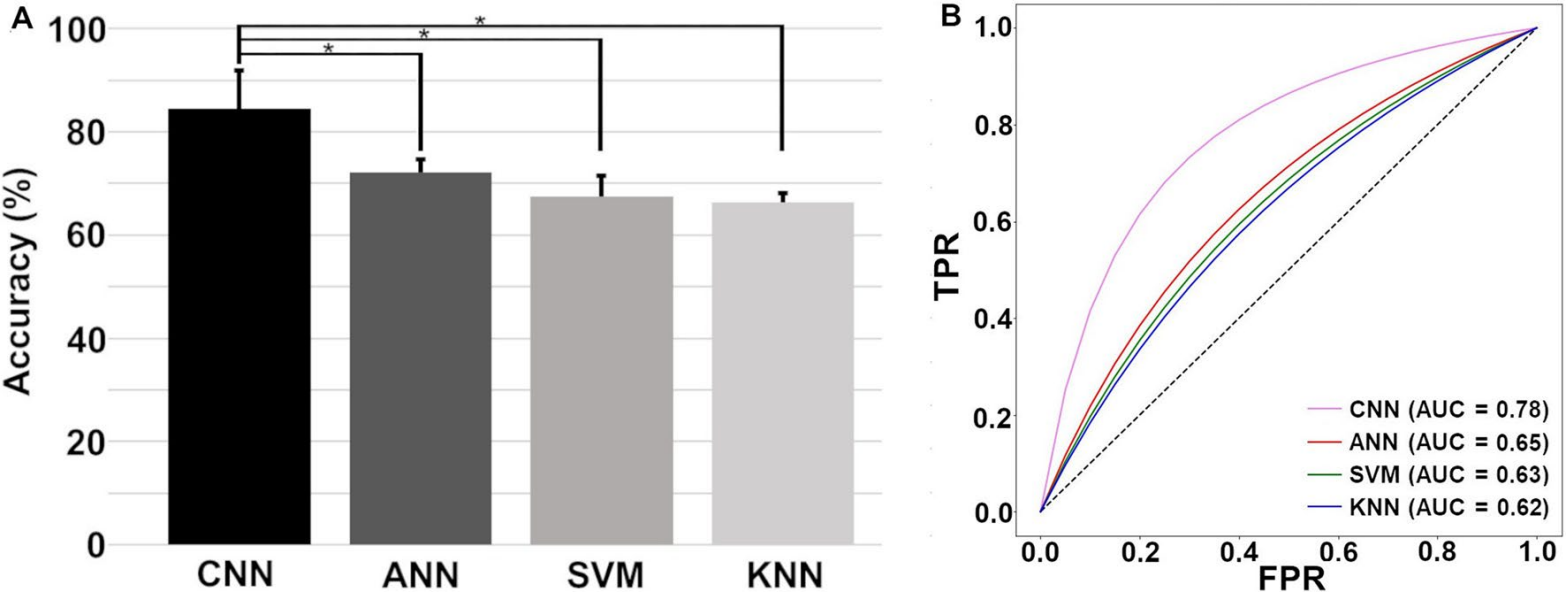
(**A**) Means and standard deviations of the prediction accuracies of each algorithm. Single asterisks (*) indicates a statistically significant difference between the prediction accuracies of different algorithms (CNN > ANN, SVM, and KNN, *p* < 0.001). (**B**) ROC AUCs (receiver operating characteristic area under curves) of CNN, ANN, SVM, and KNN to predict VTA 60 seconds before the occurrence. TPR: True Positive Rate and FPR: False Positive Rate accuracies.

